# Probe-level performance of Xpert MTB/RIF versus phenotypic culture for rifampicin resistance detection and a triage algorithm based on 3,360 clinical specimens in China

**DOI:** 10.1186/s12879-026-14037-4

**Published:** 2026-07-28

**Authors:** Yuliang Huang, Shunda Luo, Jinfeng Lu, Chaoyong He, Diyu Mai, Qiuying Ma, Wanhong Huang, Hanzhen Su, Zhengkang Li, Liang Zhang, Xiaocheng Luo

**Affiliations:** 1 Fourth People’s Hospital of Nanning, Nanning, Guangxi China; 2 Department of Clinical Laboratory, Guangdong Provincial People’s Hospital (Guangdong Academy of Medical Sciences), Southern Medical University, Guangzhou, Guangdong China; 3 Translational Medicine Center, Guangdong Women and Children Hospital, Guangzhou, Guangdong China

**Keywords:** *Mycobacterium tuberculosis*, Xpert MTB/RIF, Phenotypic drug susceptibility testing

## Abstract

**Background:**

The Xpert MTB/RIF (Xpert) assay is a cornerstone for the rapid detection of rifampicin resistance. However, discordant results between Xpert and phenotypic drug susceptibility testing (pDST) present diagnostic challenges, particularly in regions with genetically diverse *Mycobacterium tuberculosis* populations. This study aimed to evaluate the performance of Xpert at the probe level to resolve such discrepancies and to inform an optimized diagnostic workflow.

**Methods:**

Using a retrospective cohort of 3,360 clinical specimens from a high-burden setting in China, we compared Xpert results with pDST as the reference standard. We then performed a stratified analysis based on specific probe mutation patterns to determine their predictive value for phenotypic resistance.

**Results:**

Overall, Xpert demonstrated substantial agreement with pDST (*κ* = 0.722, 95% CI: 0.685–0.759), exhibiting a sensitivity of 80.4% and a specificity of 95.8%. However, concordance was highly dependent on the specific probe mutation. Mutations in Probe E were strongly predictive of phenotypic resistance (Positive Predictive Value: PPV: 91.4%). In contrast, The mutations detected by Probe ΔCt and Probe A exhibited a low PPV of only 28.0% and 14.3%, respectively. Based on these findings, we propose a diagnostic triage strategy: results indicating Probe E mutations can reliably guide immediate treatment adjustments, whereas isolated ProbeΔCt resistance, or Probe A mutations, warrant confirmatory pDST before initiating second-line regimens.

**Conclusion:**

The clinical interpretation of Xpert results requires a nuanced understanding of probe-specific mutation patterns. Our probe-stratified analysis and the resulting triage algorithm provide a practical framework for optimizing the management of rifampicin-resistant tuberculosis (RR-TB). This approach effectively balances the need for diagnostic speed with therapeutic accuracy in programmatic settings.

## Introduction

Tuberculosis (TB), caused by the *Mycobacterium tuberculosis* complex (MTBC), remains a formidable global public health challenge and a leading cause of infectious disease mortality worldwide. Despite decades of therapeutic advances, the disease continues to impose high morbidity, mortality, and a substantial economic burden, particularly in developing countries. According to the World Health Organization (WHO), there were an estimated 10.6 million new cases and 1.3 million deaths in 2022 [1–2]. The situation is further complicated by the emergence and spread of multidrug-resistant (MDR) and RR-TB strains, which result from resistance to first-line drugs like isoniazid (INH) and rifampicin (RIF) [2–4]. This escalating threat underscores the critical need for enhanced diagnostic methodologies and more effective therapeutic strategies to advance global TB control efforts.

Conventional pDST of cultured isolates remains the laboratory gold standard for diagnosing tuberculosis drug resistance, as it directly measures phenotypic resistance. However, this method is hampered by the extremely slow growth of the MTBC, resulting in a prolonged turnaround time of typically 2–4 weeks. This delay creates an urgent clinical need for rapid molecular assays that infer resistance by detecting mutations in associated genes. RIF, a key first-line anti-tuberculosis drug, acts by binding to a well-conserved 81-base pair region in the rpoB gene. This region encodes 27 amino acids (codons 507–511, Escherichia coli numbering) that form the essential binding site for RIF. Mutations within this segment can alter the conformation of the drug-binding pocket in the RNA polymerase β-subunit, reducing RIF binding affinity, impairing transcriptional inhibition, and ultimately leading to rifampicin resistance (RIF-R) [5–6]. Although the recently developed Xpert MTB/XDR assay can detect resistance to six anti-tuberculosis agents, it has not yet been adopted in routine clinical practice [7]. Conventional pDST, while more time-consuming, provides a broader resistance profile covering more than 10 drugs, including isoniazid (INH) and cycloserine (CS). However, genotypic predictions from molecular assays such as Xpert may show discrepancies when compared with the phenotypic gold standard.

This discrepancy poses a critical challenge for clinicians: whether to rely on the rapid molecular result or to await slower, culture-based confirmation—a decision with significant therapeutic implications. Multiple studies have demonstrated that Xpert shows high concordance with phenotypic culture in detecting RIF resistance, with consistently reported sensitivity and specificity. For instance, investigations across diverse geographical settings—including studies by Lavu et al. in Papua New Guinea (*n* = 884), Zetola et al. in the United States (*n* = 370), and Hussain et al. in Pakistan (*n* = 180)—have corroborated these strong performance metrics [8–12]. However, the generalizability of these findings is constrained by the limited sample sizes of culture-positive cohorts and the absence of large-scale, systematic evaluations of probe specificity to address genotype–phenotype discordance. Thus, a comprehensive probe-level assessment is warranted to resolve such inconsistencies. In this study, we analyzed 3,360 clinical cases to systematically deconstruct the performance of Xpert at the probe level. Our ultimate goal is to establish an evidence-based diagnostic triage strategy that resolves genotype–phenotype discrepancies and facilitates targeted therapeutic interventions.

## Materials and methods

### Study design and data processing

This retrospective cohort study systematically analyzed 4,843 culture-positive MTBC specimens collected from July 2023 to July 2025 at the Fourth People’s Hospital of Nanning. The specimen types included sputum (72.3%), bronchoalveolar lavage fluid (15.6%), pleural fluid (5.8%), cerebrospinal fluid (4.1%), and other body fluids (2.2%). All specimens were confirmed as MTBC-positive using the BACTEC MGIT 960 System, with species identification performed by Microflex LT mass spectrometry. The study was conducted at the Fourth People’s Hospital of Nanning, Guangxi’s tuberculosis research centre and Nanning’s quality-control hub. The hospital provides care for approximately 90,000 outpatients with suspected TB annually and admits nearly 8,000 inpatients for TB related treatment. Both Xpert testing and mycobacterial culture are routinely available.From the overall collection, a total of 3,360 consecutive cases tested for RIF resistance by both Xpert and phenotypic culture were retrospectively analyzed to evaluate the concordance between rapid molecular testing and the phenotypic gold standard in this regional setting.

The study protocol was approved by the Institutional Ethics Committee of the Fourth People’s Hospital of Nanning (Approval No. 202246). A standardized data cleaning procedure was implemented to ensure data integrity. First, duplicate records—defined as multiple specimens collected from the same patient—were identified based on patient names and ages, and only the earliest collected specimen was retained for analysis. Ambiguously matched records were further verified by cross-checking national identity numbers through the Hospital Information System (HIS). Afterwards, records with incomplete pDST outcomes or missing Xpert probe data were excluded. Ultimately, only specimens that tested positive for both Xpert and pDST were enrolled for subsequent analyses.

Phenotypic rifampicin susceptibility testing was conducted via a commercial microdilution assay with the critical concentration set at 1.0 mg/L; isolates exhibiting visible bacterial growth at concentrations ≥ 1.0 mg/L were defined as rifampicin-resistant. MTB strain H37Rv (ATCC 27294) served as the drug-susceptible control strain for every testing batch.

### Culture and pDST

Positive isolates were tested for drug susceptibility using the BACTEC MGIT 960 system with microtiter plates (Zhuhai Encode Medical Engineering Co., Ltd., China). A colorimetric method was employed to interpret results: resistance was indicated by a blue-to-red color transition, while susceptibility was defined as the retention of the original blue color. The assay included the following 13 drugs: isoniazid (INH), RIF, ethambutol (ETO), cycloserine (CS), rifabutin (RFB), ethionamide (ETO), moxifloxacin (MFX), amikacin (AM), linezolid (LZD), para-aminosalicylic acid (PAS), kanamycin (KM), clofazimine (CFZ), levofloxacin (LFX). Minimum inhibitory concentrations (MICs) were determined by the indirect proportion method, with resistance defined based on established international critical concentrations (CC).

### Xpert assay

The automated Xpert assay employs multiplex real-time PCR to amplify the 81-bp rifampicin-resistance-determining region (RRDR) of the rpoB gene. Using molecular beacon technology, the assay detects rifampicin resistance by targeting the wild-type, rifampicin-susceptible sequence of the MTBC rpoB gene. Five fluorescently labeled probes (A to E) are designed to bind distinct segments within the 81-bp core region. Hybridization of the probe to its complementary sequence generates a fluorescent signal, indicating the presence of the wild-type genotype, i.e., the susceptible genotype. Mutations within the probe-targeted region cause impaired or delayed binding, resulting in absent hybridization signals and a cycle threshold (Ct) value of zero. The Ct values also correlate with bacterial burden, where higher Ct values correspond to lower mycobacterial loads. A unique resistance pattern, termed Probe ΔCt resistance, is identified when all five probes generate valid hybridization signals, but the difference between the maximum and minimum Ct values (ΔCt) is greater than 4 [5–6]. This is caused by non-canonical site mutations in the rpoB gene. Such mutations do not completely abolish the binding of any single probe, but markedly alter the hybridization and amplification efficiency of partial probes. This results in earlier fluorescence take-off for some probes and delayed take-off for others, with the ΔCt value widening to greater than 4; accordingly, the result is interpreted as a drug-resistant mutation of the rpoB gene.

### Statistical analysis

Descriptive statistics are presented as mean ± standard deviation (*SD*) for normally distributed continuous variables, with normality confirmed by the Shapiro-Wilk test. Categorical variables are reported as counts and percentages (n, %) with 95% confidence intervals (*CIs*). The agreement between Xpert and pDST was evaluated using Cohen’s kappa (*κ*) statistic, interpreted according to established benchmarks: *κ* < 0.20, poor; 0.21–0.40, fair; 0.41–0.60, moderate; 0.61–0.80, substantial; and > 0.80, almost perfect agreement. Using pDST as the reference standard, we calculated the sensitivity, specificity, positive predictive value (PPV), and negative predictive value (NPV), each accompanied by 95% *CIs*. All statistical analyses were conducted using SPSS version 23.0, with a two-tailed α level of 0.05 defining statistical significance.

## Results

### Data filtering and baseline characteristics

**Fig. 1:**
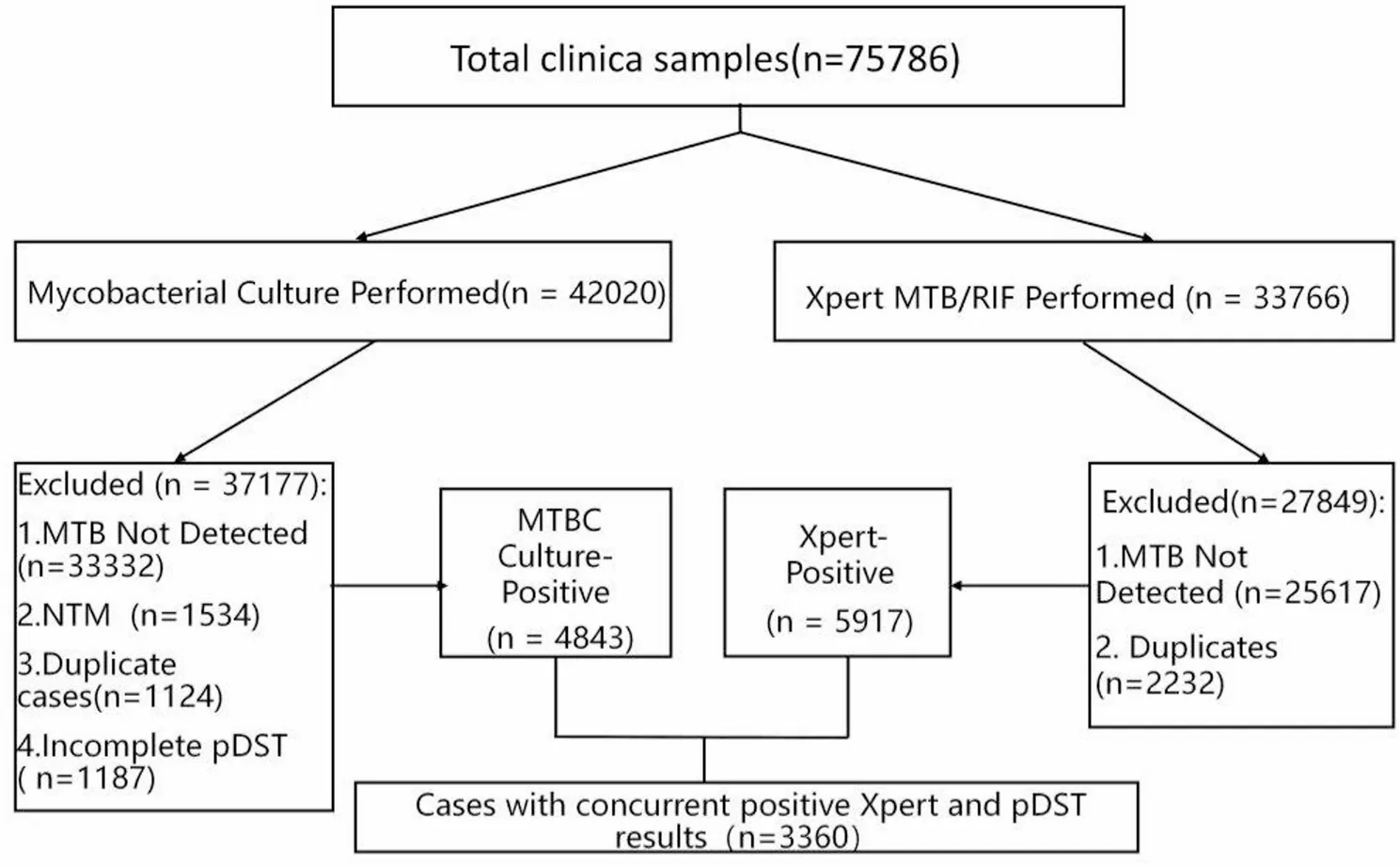
Flowchart of the participant selection process. This diagram outlines the parallel derivation of the final analysis cohort from initial clinical specimens, detailing the exclusion criteria applied along the culture-based and Xpert MTB/RIF-based pathways. MTBC: *Mycobacterium tuberculosis* complex; NTM: non-tuberculous mycobacteria; pDST: phenotypic drug susceptibility testing; RIF: rifampicin. The chart summarizes the overall sample size and screening steps for both pathways using a clear, hierarchical structure and concise wording

Following the application of exclusion criteria, 4,843 MTB isolates from unique patients, each with pDST results, were included in the study. The cohort consisted of 3,516 males (72.6%) and 1,327 females (27.4%), resulting in a male-to-female ratio of 2.65:1. The mean age of the patients was 53.48 ± 17.93 years. Sputum was the predominant specimen type (3,248 isolates, 67.1%), followed by bronchoalveolar lavage fluid (1,109 isolates, 22.9%). The final analysis cohort comprised 3,360 clinical cases with complete pDST and Xpert results (Fig. 1).

Resistance patterns were analyzed for 13 anti-tuberculosis agents, with a focus on the following:

1. Combined resistance profiles of the first line drugs isoniazid (INH) and rifampicin (RIF) alongside other agents;
2. Cross resistance patterns among fluoroquinolones (FQs) within second line drugs;
3. The distribution of co resistance in: Hr TB (isoniazid monoresistant, rifampicin susceptible tuberculosis), MDR TB (multidrug resistant tuberculosis, resistant to both INH and RIF), and Pre XDR/XDR TB (MDR TB with additional resistance to FQs or second line injectable agents).

### Overall concordance between Xpert and pDST for rifampicin resistance

The analysis included 3,360 patients with MTB infection confirmed by both Xpert and culture. pDST identified 2,973 RIF-susceptible isolates (88.5%) and 387 RIF-resistant isolates (11.5%). In comparison, the Xpert assay detected 2,924 susceptible (87.0%) and 436 resistant (13.0%) cases. Using pDST as the reference standard, the Xpert assay demonstrated a sensitivity of 80.4% (311/387) and a specificity of 95.8% (2,848/2,973). The PPV was 71.3% (311/436), and the NPV was 97.4% (2,848/2,924). The overall agreement between the two methods was substantial (Cohen’s *κ* = 0.722, 95% CI: 0.685–0.759). Despite this agreement, a statistically significant discrepancy in rifampicin resistance classification was observed (*χ² =1758.75, P < 0.001*; Table 1).

**Table 1:** Performance of Xpert for rifampicin resistance diagnosis against pDST as the reference standard

Test	pDST *R* (*n*)	pDST S (*n*)	Sensitivity % (95% CI)	Specificity % (95% CI)	PPV % (95% CI)	NPV % (95% CI)	Kappa (κ)
Xpert R	311	125	80.4 (76.2–84.2)	95.8 (94.7–96.7)	71.3 (66.8–75.5)	97.4 (96.6–98.1)	0.722
Xpert S	76	2848					

pDST: phenotypic drug-susceptibility testing; R, rifampicin-resistant; S, rifampicin-susceptible; PPV: positive predictive value; NPV: negative predictive value

### Probe-level stratification reveals heterogeneity in phenotypic resistance prediction

A probe-specific analysis was performed on 436 Xpert positive, RIF-resistant cases with corresponding pDST results. The analysis revealed distinct phenotypic concordance profiles associated with different mutation patterns. Single-probe mutations, which constituted the most prevalent category (84.4%, 368/436), were associated with phenotypic resistance in 76.6% of cases (282/368), while 23.4% (91/368) were phenotypically susceptible. In contrast, mutations detected specifically by Probe A and Probe ΔCt yielded low PPV, at 11.5% (7/49) and 28% (14/50), respectively. However, these patterns were associated with high NPV of 85.7% and 72.0% **(**Table 2**).**

**Table 2:** Association between Xpert-detected rpoB mutations and phenotypic rifampicin resistance

Mutation probe	Total (*N*)	pDST					
		*R* (n1)	n1/*N* (PPV, %)	95% CI (%)	S (n2)	n2/*N* (NPV, %)	95% CI (%)
A	49	7	14.3	5.9–27.2	42	85.7	72.8–94.1
B	20	15	75.0	50.9–91.3	5	25.0	8.7–49.1
C	8	5	62.5	24.5–91.5	3	37.5	8.5–75.5
D	67	50	74.6	62.5–84.5	17	25.4	15.5–37.5
E	224	205	91.5	87.1–94.8	19	8.5	5.2–12.9
ProbeΔCt	50	14	28.0	16.2–42.5	36	72.0	57.5–83.8
AB	7	6	85.7	42.1–99.6	1	14.3	0.4–57.9
AD	4	3	75.0	19.4–99.4	1	25.0	0.6–80.6
AE	1	1	100.0	2.5–100.0	0	0.0	0.0–97.5
BE	2	2	100.0	15.8–100.0	0	0.0	0.0–84.2
DE	4	3	75.0	19.4–99.4	1	25.0	0.6–80.6

RIF: rifampicin; R: rifampicin-resistant; S: rifampicin-susceptible; pDST: phenotypic drug-susceptibility testing; 95% CI: 95% confidence interval

### Optimized diagnostic performance following exclusion of high-interference probe signals

After excluding isolates with isolated Probe A and ProbeΔCt mutation patterns, the performance of the Xpert assay was re-evaluated. McNemar’s test with continuity correction confirmed a statistically significant systematic discrepancy between Xpert and pDST results (*χ²* = 2084.37, *P* < 0.001), reflecting an imbalance in the distribution of false positives and false negatives rather than random agreement. Despite this systematic difference, the overall agreement between the two methods was excellent (Cohen’s *κ* = 0.802, 95% CI: 0.767–0.837). The optimized Xpert assay achieved a sensitivity of 78.7% (95% *CI*: 74.2–82.7%), specificity of 98.4% (95% *CI*: 97.8–98.9%), PPV of 86.2% (95% *CI*: 82.0–89.6%), and NPV of 97.4% (95% *CI*: 96.6–98.1%) against the pDST gold standard **(**Table 3**).**

**Table 3:** Diagnostic performance of Xpert MTB/RIF for rifampicin susceptibility testing after excluding Probe A and ProbeΔCt

Test	pDST *R* (*n*)	pDST S (*n*)	Sensitivity % (95% CI)	Specificity % (95% CI)	PPV % (95% CI)	NPV % (95% CI)	Kappa (κ)
Xpert R (excl. Probe A & ProbeΔCt)	280	45	78.7 (74.2–82.7)	98.4 (97.8–98.9)	86.2 (82.0-89.6)	97.4 (96.6–98.1)	0.802
Xpert S (excl. Probe A & ProbeΔCt)	76	2848					

pDST: phenotypic drug-susceptibility testing; R, rifampicin-resistant; S, rifampicin-susceptible; PPV: positive predictive value; NPV: negative predictive value;κ ≥ 0.8 indicates almost perfect agreement

### A probe-level diagnostic triage algorithm for clinical management

**Fig. 2:**
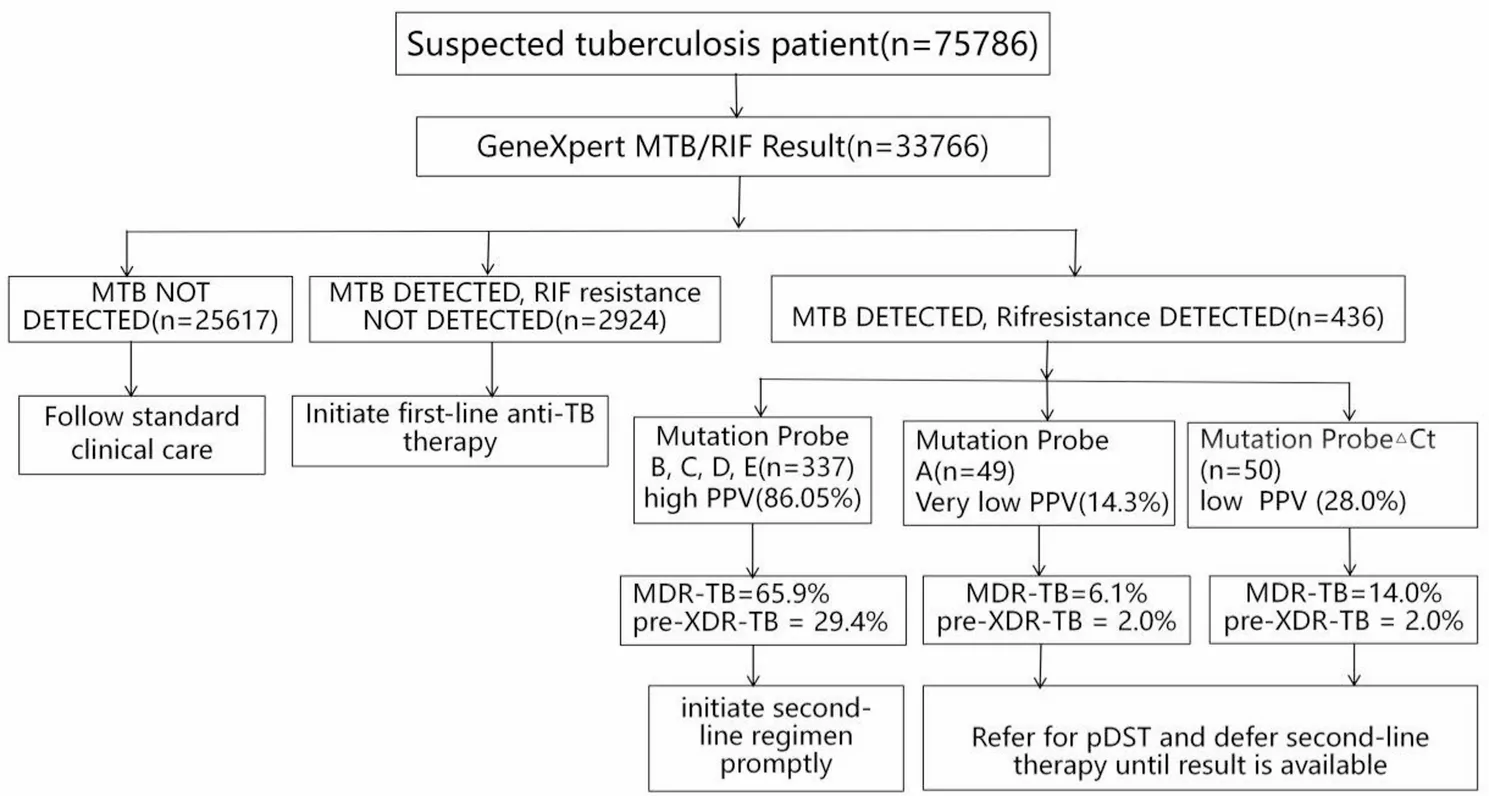
Diagnostic triage algorithm outlining the pathway from probe mutation analysis to clinical decision

Given the substantial differences in PPV among various probe mutations **(**Table 2 and Table 3**)**, we developed a pragmatic diagnostic triage algorithm **(**Fig. 2**).** For specimens where Xpert indicates rifampicin resistance through mutations in Probes B, C, D, or E—which collectively demonstrate a high PPV 86.05%—Out of the 337 isolates with mutations in Probes B/C/D/E, 65.9% (222/337) exhibited MDR-TB, characterized by resistance to both RIF and INH. The likelihood of pre-XDR-TB and XDR-TB stood at 29.4% each (99/337), underscoring the substantial presence of isolates with resistance to both FQs and second-line injectable agents within this high-risk subset. In contrast, the PPV for samples classified as rifampicin resistance using the Probe A method and the Probe ΔCt method were 14.3% (7/49) and 28.0% (14/50), respectively. The proportions of MDR-TB resistance identified by the two methods were 6.1% (3/49) and 14.0%(7/50), while the proportions of pre-XDR-TB were both 2.0%.

### Comprehensive drug resistance profile from pDST

Among 4,843 MTB isolates analyzed, 2,727 (56.3%) were classified as drug-susceptible, while 2,116 (43.7%) were resistant to at least one anti-tuberculosis agent. The resistance profiles revealed distinct patterns: resistance to CFZ was most prevalent (18.1%, 879 isolates), followed by INH (15.3%, 743 isolates), Cs (13.9%, 675 isolates), and RIF (10.0%, 482 isolates). Notably, resistance to FQs (KM/MFX/AM) was observed in 7.2% (348 isolates) of cases. Further stratification of resistance patterns identified 409 isolates (8.4%) with Hr-TB, 334 isolates (6.9%) with MDR-TB, and 141 isolates (2.9%) with pre-XDR or XDR-TB.

The resistance rates to the 14 anti-tuberculosis agents, in descending order, were as follows: CFZ (18.15%) > INH (15.28%) > Cs (13.94%) > RIF (9.95%) > RFB (6.42%) > LFX (6.03%) > MFX (5.82%) > PAS (4.65%) > LZD (4.19%) > KM (3.88%) > EMB (2.33%) > AM (1.61%) > ETO (1.51%).

pDST was performed using the BACTEC MGIT 960 system with standard critical concentrations **(**Table 4**).**

**Table 4:** Resistance profiles of mycobacterium tuberculosis to 13 antituberculosis drugs

Total (*N* = 4843)	Resistant strain (*n*)	*n*/*N* (%)	95% CI (%)
Drug	2116	43.80	42.3–45.3
Isoniazid, H	740	15.28	14.3–16.3
Rifampicin, R	482	9.95	9.1–10.9
Rifabutin, RFB	311	6.42	5.8–7.1
Ethambutol, E	108	2.23	1.8–2.7
Cycloserine, Cs	675	13.94	13.0-14.9
Kanamycin, KM	188	3.88	3.4–4.5
Amikacin, AM	78	1.61	1.3-2.0
Linezolid, L	203	4.19	3.7–4.8
Levofloxacin, LFX	292	6.03	5.4–6.7
Moxifloxacin, MFX	282	5.82	5.2–6.5
Ethionamide, ETO	75	1.55	1.2–1.9
Para-aminosalicylic, PAS	225	4.65	4.1–5.2
Clofazimine, CFZ	879	18.15	17.1–19.2

Among isoniazid-resistant isolates, 50.6% co-developed RIF-resistance (MDR-TB) and 28.8% acquired FQ resistance (pre-/XDR-TB)

Among RIF-resistant isolates, 69.3% exhibited concurrent isoniazid resistance (MDR-TB), while 31.7% showed FQ resistance (pre-/XDR-TB)

## Discussion

The rising threat of drug-resistant MTBC poses a major challenge to global public health. Achieving the goals of the WHO’s End TB Strategy necessitates effective active case-finding and, crucially, rapid and accurate diagnosis for optimal patient management and disease control. The Xpert test, a molecular diagnostic tool endorsed by the WHO, offers significant advantages over traditional pDST, including the ability to detect RIF-resistance within two hours [13–14]. Our large-scale analysis of 3,360 clinical specimens confirms that the Xpert assay is a reliable frontline screening tool for RIF resistance, demonstrating substantial overall concordance with pDST. A key finding is that the clinical predictive value of a “RIF-resistant” result is highly heterogeneous and depends on the specific probe mutation pattern. Consequently, while Xpert remains an indispensable rapid screening tool, our data unequivocally indicate that pDST confirmation is essential for a definitive diagnosis, particularly in cases with these specific mutations. These findings underscore the necessity of an integrated diagnostic workflow that synergizes molecular rapidity with phenotypic confirmation.

Notably, despite the high overall agreement between Xpert and pDST observed in this study, the molecular assay cannot fully replace culture-based pDST. First, Xpert only targets mutations within the 81-bp RRDR of the rpoB gene and fails to detect variants outside this region. Furthermore, even when restricted exclusively to mutations located within this 81-bp RRDR, a statistically significant discordance between Xpert and pDST was still observed in the interpretation of rifampicin resistance (*P < 0.001*). Among 2,924 cases classified as susceptible by Xpert, pDST identified 76 (2.6%) as resistant. Conversely, of the 436 cases detected as resistant by Xpert, pDST categorized 125 (28.7%) as susceptible. Using pDST as the reference standard, the diagnostic performance of the Xpert assay was as follows: sensitivity, 80.4%; specificity, 95.8%; PPV, 71.3%; and NPV, 97.4%. The high NPV supports the utility of Xpert for rapidly ruling out RIF-resistant, particularly in emergency and high-risk populations, thereby enabling prompt initiation of appropriate tuberculosis treatment in patients with no detectable resistance mutations. We attribute the observed discordance to several factors: (1) There are different levels of drug resistance conferred by distinct mutation sites in the rpoB gene [15–16]. For mutations with weak resistance phenotypes, Xpert is capable of detecting these variants, while pDST still classifies the isolate as wild-type and susceptible. The mutation site targeted by Probe A may belong to such weakly resistant loci, thereby resulting in a lower specificity of mutation detection for Probe A. (2) Heteroresistance: In Xpert assay, the rpoB probes are perfectly matched to the wild-type susceptible sequences. For clinical samples containing a mixed population of wild-type susceptible MTB and RIF-resistant mutant strains, when the proportion of wild-type susceptible strains exceeds 35%-40%, the probe signals show no obvious reduction, and Xpert interprets the result as wild-type susceptible. Nevertheless, the resistant mutant strains present in the sample can test positive for resistance by pDST. Namely, the Xpert assay produces false-negative interpretations [11]. (3) Silent or compensatory mutations: Polymorphisms outside the rifampicin resistance-determining region may be flagged by Xpert probes without conferring actual phenotypic resistance [10, 17], Therefore, such mutation detections by Xpert are regarded as false positives. (4) Factors that reduce PCR efficiency, such as low bacterial load and PCR inhibition, may abolish probe hybridization signals and further lead to false-positive detection of drug-resistant mutations [18–19].

Our probe-stratified analysis of 436 Xpert-positive cases revealed significant heterogeneity in clinical reliability at the individual probe level, underscoring the need for a more nuanced interpretation of “RIF-resistant” results in clinical practice. Specifically, mutations identified by Probes B, C, D, and E demonstrated high PPV (> 85%) for phenotypic resistance. In contrast, results based solely on a Probe A mutation showed a markedly low PPV of14.3% corresponding to a 72% false-positive rate in our cohort.

Probe A detects mutations at codons 507–511 of the rpoB gene (Escherichia coli numbering), corresponding to the extreme 5′end of the 81-bp RRDR region. This region contains frequent low-level resistance mutations as well as mutations unrelated to drug resistance, both of which often yield Xpert-positive but pDST-susceptible result [17, 20]. Post-hoc optimization of Xpert performance, excluding isolates with isolated Probe A or ProbeΔCt patterns, further illustrated the critical trade-off between reducing misclassification and minimizing missed RIF-resistant (RIF-R) cases in real-world practice. While this exclusion strategy reduced false positives (125 remaining in our cohort of 3360 isolates) and improved overall PPV to 86.2%, it came at the cost of reduced sensitivity (78.7%), with 76 true RIF-R cases classified as false negatives. This balance is particularly salient for high-burden regions: fewer false positives streamline treatment pathways, reduce unnecessary second-line therapy exposure, and contain healthcare costs, yet missed RIF-R cases pose risks of delayed appropriate treatment and ongoing transmission if not confirmed by pDST.

In alignment with the 2022 WHO stepwise diagnostic algorithm [21, 22], we propose a molecular triage and phenotype-directed cascade strategy tailored for high-burden settings. This approach leverages the rapidity of the Xpert assay for same-day triage: high-confidence mutations, such as those identified by Probe E, may warrant an immediate sitch to a second-line treatment regimen. Conversely, specimens yielding ambiguous signals, particularly singular mutations detected by Probe A, should not lead to empirical regimen adjustment unless confirmed by pDST. This algorithm effectively harmonizes rapid diagnosis with precise treatment, mitigating the risk of unnecessary treatment while promptly pinpointing genuine drug-resistant cases. Excluding Probe A may reduce false positives associated with the Probe ΔCt resistance pattern, but it could also lead to missed detection of some RIF-resistant cases. Its tiered design is particularly suitable for resource-constrained settings where Xpert testing has already been scaled up, but phenotypic culture capacity remains limited.

If Xpert identifies RIF resistance and initiates second-line drug treatment as usual, the cost per individual amounts to $18,000. Nonetheless, in the probe-based diversion approach investigated in this study, 17.9% (78/436) (Table 2) of the drug-resistant cases identified by Probe ΔCt and Probe A were susceptible to RIF. The cost of RIF therapy per person stood at $800, representing a $17,200 reduction compared to second-line drug therapy. Consequently, the diversion tactic resulted in a collective saving of 17.1% in treatment expenses [23–24].

pDST revealed a distinct hierarchy of resistance (Table 3), with CFZ exhibiting the highest resistance rate (18.9%), followed by INH (15.3%), CS (13.9%), and RIF (10.0%). A critical finding was that 69.3% of RIF-resistant isolates also showed INH resistance (corresponding to an MDR-TB risk of 50.6%), and among these, 31.7% exhibited additional FQ resistance (indicating a potential XDR-TB risk). Systematic stratification identified three predominant resistance profiles: (1) 409 isolates (8.4%) with monoresistance to INH (Hr-TB), (2) 558 isolates (6.9%) with MDR-TB, and (3) 141 isolates (2.9%) with pre-XDR or XDR-TB. These findings advocate for a shift from empirical treatment toward precision medicine, in which therapeutic regimens are iteratively tailored according to comprehensive pDST profiles, thereby improving treatment efficacy and reducing the risk of failure in drug-resistant TB management. The prevalence of RIF and INH resistance observed in our cohort was lower than previously reported [25]. This discrepancy may be attributed to geographical heterogeneity, as MTB isolates from different provinces in China exhibit distinct resistance patterns. Furthermore, our hospital functions as a primary screening center; most participants were treatment-naïve, which inherently results in a lower proportion of drug-resistant isolates. Although culture-based pDST provides a comprehensive resistance profile, its long turnaround time remains a limitation. There is a corresponding need for rapid molecular assays to assess susceptibility to other anti-tuberculosis agents. The XDR-LFC probe, for instance, enables rapid XDR-TB screening directly from sputum samples by detecting resistance-conferring mutations across seven MTB genes—covering six drug classes, including INH, RIF, and FQs—and has demonstrated superior performance over whole-genome sequencing (WGS) in identifying relevant drug-resistance mutations [26–27]. For patients with sputum-scarce pulmonary tuberculosis, who are often underrepresented in conventional drug resistance surveillance, nanopore sequencing using bronchoalveolar lavage fluid offers an alternative strategy with favorable diagnostic accuracy [28]. Nonetheless, sustained research and technological innovation are essential to further address the challenges of drug-resistant tuberculosis.

This study has several notable limitations. First, this was a single-centre retrospective analysis. Although our proposed “probe information triage algorithm” constitutes a conceptually reasonable data-driven framework, it was developed solely on retrospective datasets; therefore, additional external and prospective experimental validations are needed to verify its performance. Second, we only included 3360 specimens with concurrent positive Xpert and culture-based pDST results to assess the concordance between molecular and phenotypic drug resistance, which may induce selection bias affecting our conclusions. Xpert detects MTB via nucleic acid amplification independent of bacillary viability at sampling, whereas culture-based pDST relies on viable mycobacteria and is highly dependent on bacterial activity at specimen collection. A subset of patients had received prior anti-tuberculosis treatment before sampling. Among patients harbouring drug-susceptible MTB, prior therapy may eliminate viable bacilli and lead to negative culture results, even though Xpert can still detect mycobacterial nucleic acids. Restricting our analysis to dual-positive specimens inherently enriches phenotypically resistant isolates within our cohort and overrepresents resistant strains in pDST outcomes. Finally, the low positive predictive value (PPV) of Probe A and Probe ΔCt reported herein was defined exclusively by probe reaction signals. For discordant specimens that were predicted resistant by Probe A and Probe ΔCt but confirmed susceptible by culture, we did not conduct targeted sequencing of rpoB resistance-determining regions or whole-genome sequencing of MTB isolates to elucidate the mechanisms behind these mismatched results.

## Conclusion

Based on the substantial cohort sizes reported to date, our findings illustrate that although Xpert shows good concordance with pDST, the clinical implication of a RIF-resistant result is heavily dependent on the performance of specific probes. Resistance mutations detected via Probe ΔCt and Probe A exhibit a low PPV. The Xpert probe-based triage algorithm for MTB drug resistance detection proposed in this study is conducive to optimizing the clinical application of Xpert. Further prospective, multicenter studies are still required to validate the reliability and generalizability of this novel triage strategy.

## Data Availability

The datasets used and/or analyzed during the current study are available from the corresponding author on reasonable request.

## Abbreviations

AM: Amikacin
CFZ: Clofazimine
CS: Cycloserine
Ct: Cycle threshold
EMB: Ethambutol
ETO: Ethionamide
FQ: Fluoroquinolone
INH: Isoniazid
Hr-TB: Isoniazid-resistant tuberculosis
HIS: Health information system
INH: Isoniazid
KM: Kanamycin
LFX: Levofloxacin
LZD: Linezolid
MDR-TB: Multidrug-resistant tuberculosis
MICs: Minimum inhibitory concentrations
MFX: Moxifloxacin
MTBC: Mycobacterium tuberculosis complex
NPV: Negative predictive value
PAS: Para-aminosalicylic acid
PPV: Positive predictive value
pre-XDR-TB: Pre-extensively drug-resistant tuberculosis
RIF: Rifampicin
RFB: Rifabutin
RIF-resistant: Rifampicin-resistant
RR-TB: Rifampicin-resistant tuberculosis
RRDR: Rifampicin resistance-determining region
SM: Streptomycin
SD: Standard deviation
RIF-S TB: RIF-susceptible tuberculosis
TB: Tuberculosis
WHO: World Health Organization
Xpert: GeneXpert MTB/RIF
